# Development of the Nursing Relationships Scale: a measure of interpersonal approaches in nursing care

**DOI:** 10.1186/1752-4458-4-12

**Published:** 2010-05-28

**Authors:** Tan Kan Ku, Harry Minas

**Affiliations:** 1 Discipline of Nursing and Midwifery, School of Health Sciences, RMIT University, 330 Swanston Street, Melbourne VIC 3000, Australia; 2 Centre for International Mental Health, Melbourne School of Population Health, The University of Melbourne, Parkville, Victoria 3010, Australia

## Abstract

**Background:**

There is no comprehensive measure of dimensions describing the nursing relationship that is suitable for use with survey samples and that is focused on nursing particular types of patients. The objective of this study was to develop a measure to investigate significant dimensions of the nurse-patient relationship, the Nursing Relationship Scale (NRS).

**Methods:**

Hypothetical cases (diabetes or mental illness) in vignette format were presented to 132 psychiatric and 76 general nurses. Thirty-four questions about the nurse-patient interaction were asked. Principal component analyses (with oblique rotation) were used to identify underlying dimensionality in the correlations of items, combining ratings from the two case vignettes. Scales were constructed from the final solution and Cronbach's alpha coefficients calculated. Subscale score variations were analysed across nurse type and patient type to examine the discriminant validity of the subscales.

**Results:**

Principal components analysis revealed five dimensions accounting for 52 percent of the variation within items. Four 'conceptual' factors were derived. These were labeled Caring/Supportive Approach, Nursing Satisfaction, Authoritarian Stance, and Negativity. Developed as subscales, reliability analysis indicated high internal consistency with respective alpha coefficients for the diabetes case 0.91, 0.75, 0.65, and 0.78 and for the mental illness case of 0.91, 0.75, 0.73, and 0.85. There was significant variation in scale scores according to nurse type (psychiatric versus general) and patient type (diabetes versus mental illness). Nurses endorsed more highly items from the subscales Caring/Supportive Approach and Nursing Satisfaction than items from Authoritarian Stance (with intermediate endorsement) and Negativity (lowest endorsement) subscales.

**Conclusions:**

Psychometric evaluation of the NRS suggests it is a reliable instrument for measuring four key dimensions of the nurse-patient relationship and enables the study of this relationship in large samples.

## Background

For research in health care delivery to be relevant to the needs of communities, it must be sensitive to pertinent cultural aspects of those communities. There is a need to promote cultural awareness among health professionals to enable them to improve their confidence and skills in promoting holistic care to patients from diverse cultural backgrounds. An important consideration is the nature and quality of the interaction between health personnel and the patient, who may be from different cultural backgrounds. This is particularly important in multicultural societies such as Australia. Cultural diversity poses many challenges in the provision of mental health services [1-9]. In the health care delivery system nurses are the most numerous professional group and, in comparison with doctors, social workers, psychologists and occupational therapists, spend the largest amount of time in direct contact with patients. They therefore have considerable opportunity to influence patients' attitudes and behaviours in relation to their treatment, rehabilitation and recovery process.

As nursing requires a focus on therapeutic interaction between the nurse and the patient, it is likely that the attitudes and interpersonal practices the nurse brings to this interaction will influence his/her care of the patient. It is potentially useful to study the nurse/patient interaction by measuring aspects of this interaction through the use of an instrument that specifically examines nursing approach towards a particular type of patient. Improved understanding of interpersonal attitudes and practices will enhance the ability of nurses to provide problem-focused care that is appropriate to the patient.

Specific nursing knowledge, techniques and procedures are enacted through the medium of interpersonal interaction between the nurse, the patient and often the patient's social network (especially caregivers and family). Within this relationship nurses communicate a wide range of information including health and treatment related issues. However, the relationship is also a vehicle for the development of patient trust, comfort, sense of being respected and involvement in the management of the illness. These may be of equal importance to the experience of a positive therapeutic contact by patients and family [10-12]. The Edinburgh Caring Dimensions Inventory (CDI), [13] was developed on the basis of a wide examination of the empirical literature on nursing care. The focus of measurement of the CDI is on general nursing care perceptions of nurse practitioners. The CDI contains a number of items that measure the interpersonal encounter (e.g., '*getting to know the patient as a person*'). However, it also measures the application of instrumental nursing actions (e.g., 'measuring the vital signs of the patient') as well as tasks unrelated to direct care (e.g., 'making a nursing record about the patient'). The focus of the CDI is on exploring the broad concept of nursing care. Lea, Watson and Deary [14] reported that two major dimensions measured by the CDI are 'psychosocial' (of interest to the development of the NRS) and 'professional/technical' aspects of nursing care. Lea and Watson [15] showed that psychosocial elements were invariant across nursing in different settings (surgical versus general medical wards) while showing differences between settings along the 'professional/technical' dimension. Caris-Verhallen [16] developed the Questionnaire for Nurses Working in Elderly Care (QNWEC) with relevant measurement scales. The QNWEC was developed from a theoretical basis covering a series of care dimensions including friendliness, showing personal concern, taking time with the patient, maintaining a rapport, among others. However in the original work, Caris-Verhallen [16] applied this scale to only 47 nurses and provided little in the way of a psychometric analysis of the empirical dimensionality underlying the item scales. Only Cronbach's alpha coefficients were reported for the overall scale. While total scale alphas were high (for ratings of content importance = 0.84; for ratings of content experience = 0.89) this does not necessarily mean that the scale is unidimensional [17]. In addition, both the CDI and QNWEC measure a range of positive nursing actions but omit measurement of undesirable qualities such as negativism towards the patient or practice and personal barriers.

During the design of a study of cultural factors and level of contact with the mentally ill, and how these might affect overall nursing approach [18], we were surprised by the absence in the literature of an instrument capable of measuring significant interpersonal factors in the nurse-patient relationship from the point of view of the nurse. The Nursing Relationship Scale (NRS) was developed to fill this gap. While there has been significant exploration of the nurse-patient interaction using qualitative methods (e.g., [19]) there appears to be less work using closed-ended questionnaires [14], which constitute a more versatile approach when gathering information from large samples using survey methods. In this paper we report the development of a new measure, the Nursing Relationship Scale (NRS). Ethics approval was obtained from the University of Melbourne Human Research Ethics Committee.

## Methods

### Subjects

Two hundred and eight (208) nurses participated in this study. They were selected for the purpose of a broader study on the influence of cultural factors in nursing practice within psychiatric and general nursing settings. As a result the sample was comprised of 49 Chinese-Australian and 83 Anglo-Australian psychiatric nurses, and 35 Chinese-Australian and 41 Anglo-Australian general nurses. One hundred and forty eight (148) nurses were women and 60 were men. The mean age of the sample was 44.8 years (s.d. = 9.6), ranging from 21 to 65 years of age. For the overall sample the mean number of years working in a psychiatric setting was 9.8 (s.d. = 11.0) and the mean number of years in a general setting 11.2 (s.d. = 12.0). Table 1 shows the demographics of the sample.

**Table 1:** Sample Demographics (N = 208)

	Chinese- Australian	Chinese- Austrailan	Anglo- Australian	Anglo- Australian	χ^2^/F Value
	Psychiatric Nurse	General Nurse	Psychiatric Nurse	General Nurse	
	(n = 49)	(n = 35)	(n = 83)	(n = 41)	
**Age **^#^(mean, s.d.)	50.3 (5.8)	47.9 (6.0)	43.1(9.6)	39.5 (11.6)	13.2*** F(1,197)

**Sex (**male/female)	25/24	1/34	31/52	3/38	35.4***

**Years in mental health nursing **(mean, s.d.)	19.5 (10.4)	0.03 (0.1)	12.9 (9.2)	0.07 (0.2)	68.8*** F(1,204)

**Years in general nursing **(mean, s.d.)	7.7 (10.5)	23.1 (9.0)	6.1 (9.3)	15.6 (12.5)	27.3***

# Seven (7) missing cases are excluded from the analysis.

*** p < .001.

For all χ^2 ^analysis, df = 3

### Item Development of the NRS

Thirty-four items (Additional File 1) were constructed, some of which were drawn from Caris-Verhallen [16] and Watson and Lea [13]. Items construction took into account general and psychiatric settings, the latter of which may involve more restrictive or controlling attitudes in relation to the patient's behaviour (e.g., *If Mr J/S refuses medication I would try to enforce 'doctor's orders*'), derived from Ku's (2008) nursing experience. The overall content of the questionnaire includes information provision (e.g., *I would take care, more than usual, to provide Mr J/S with an explanation about a nursing action or treatment*), encouragement of communication (e.g., *I would take care, more than usual, to ask Mr J/S about his state of health*), concerns about patient's behaviour (e.g., *I would worry, more than usual, that Mr J/S may become aggressive in the ward*), emotional avoidance (e.g., *I would feel a 'barrier' between me and Mr J/S, more so than other patients*), behavioural avoidance (e.g., *I would be reluctant to work together with Mr J/S to develop a care plan*), perceived difficulty in patient management (e.g., *I would expect that Mr J/S would be a more demanding patient than most*), perceived inability in the patient (e.g., *I would have some doubt that Mr J/S could contribute significantly to his care plan*), behaviour towards caregivers and others (e.g., *Compared with other patients, I would be very supportive to the caregivers of Mr J/S*). The NRS ratings focus on a particular patient and a number of items are negatively worded (e.g., *Working with Mr J/S would be monotonous and too routine versus Looking after Mr J/S could be a challenge that I look forward to*). Responses are on a five-point bi-directional scale with the following descriptions: *disagree, tend to disagree, neither, tend to agree, agree*. For many items, the respondent is invited to consider the specific patient described in the vignette in comparison with patients in general, using phrases such as '*than other patients in the ward', 'compared with other patients' *and '*more so than usual'. *The items measure particular efforts, attitudes and actions in relation to the patient (vignette) being rated. Care was taken in constructing items, particularly those measuring undesirable characteristics, so that they were not worded in the extreme form. For example, the item '*I would not trust that Mr J/S could contribute to his care plan*' was changed to '*I would not completely trust that Mr J/S could contribute significantly to his care plan*'. This was undertaken to reduce the impact of social desirability, which often results in reduction in item variance by skewing responses towards one end of the scale.

In the present application the NRS was administered with reference to two hypothetical cases, presented as vignettes (Appendix 1). The first case describes Mr Jones who is suffering from diabetes and the second case Mr Smith who is suffering from a mental disorder. Case vignettes were constructed to highlight psychosocial disability and uncertainty regarding the potential for interpersonal aggression.

### Procedure

After ethics approval for the study was granted by the University of Melbourne Human Research Ethics Committee (HREC No. 020030), nurses were invited to participate and the purpose and nature of the study were explained. Because general and psychiatric nurses, and Anglo-Australian and Chinese-Australian nurses, were required for the study recruitment was achieved through a snowballing method. An initial pool of nurses (n = 20), working in different institutions and of different cultural backgrounds, was identified to be asked to participate in the study and to provide access to other nurses.

Nurses in the initial pool known to the researcher were asked to talk with other potential participants and ask permission for the researcher to approach them to introduce the study formally. Those nurses who expressed an interest in participating were invited to meet with the researcher for the purpose of further explanation of the nature, purpose and procedures of the study. All participants signed a written consent to anonymous participation. Data were collected in the latter part of 2002 and early 2003. The diabetes vignette preceding the mental illness vignette (of the same questionnaire) was given to the nurses to score their responses.

### Response Rate

Three hundred and forty seven (347) questionnaires were distributed either in person or by mail after initial contact with the prospective participants by Ku [18]. Two hundred and twenty four were returned (response rate of 64.6%). 16 of the completed questionnaires were excluded from analysis because they came from non-Anglo-Australian and non-Chinese-Australian nurses. Of the 331 potential participants who were Anglo-Australian or Chinese-Australian 208 returned completed questionnaires, a response rate of 62.8%.

### Statistical Analysis

Principal component analyses were used to identify common dimensions underlying the variation of item scores of the NRS. Cronbach's alpha coefficients were calculated to estimate the internal reliability of the derived NRS subscales. Two factors (nurse type and patient type) analyses of variance with repeated measures on patient type were used to examine the discriminant validity of the subscales. T-tests were used to compare any two groups on a dependent variable or to compare within sample pairwise differences on responses to different NRS subscales. All analyses were conducted using the Statistical Package for the Social Science (SPSS Version 11).

## Results

### Dimensions of the NRS

A number of preliminary principal components analyses were conducted to explore the dimensionality of the NRS within each patient type - diabetes and mental illness. This exploration revealed generally similar factors with nine factors having eigenvalues greater than or equal to one. Scree tests indicated that three, four or five factor solutions were reasonable to examine further for their coherence in content. To incorporate cross-patient type variation in addition to within-patient type variation both patient type responses were factor analysed together. A five-factor structure on the basis of the Scree plot appeared to be optimal. Factors were rotated obliquely to allow an examination of their interdependence and to help interpret loadings. Item composition was then examined for consistency in item loadings. Here the one concern was to see if corresponding items from the two patient types would load on the same factor. For the majority of items this was the case for the first three factors while factors four and five appeared to be the same in content but reflected ratings of the diabetes patient and mental illness patient respectively. Thus, conceptually there were four dimensions. Any items from the two patient types that did not load on the same factor or did not load on the corresponding fourth or fifth factor were removed from the analysis. Typically these had low communalities and loadings on the various factors.

After removal of these items principal components analysis led to the solution shown in Table 2. The five factors together accounted for 52 percent of the variance in item scores. Examination of the correlation matrix between factors revealed low values (ranging from 0.00 and 0.34) suggesting their relative independence. The highest correlation (negative) was between Factor 5 and Factor 1 (r = -0.34). The four 'conceptual' dimensions reflect the following constructs: Caring/Supportive Approach (CARE) (the tendency to spend time explaining relevant issues, taking extra care, providing encouragement, and, giving more care and treatment explanation); Nursing Satisfaction (SATI) (perceptions that the nursing of this patient is both challenging and satisfying); Authoritarian Stance (AUTH) (taking control of the management regardless of the patient's involvement), and Negativity (NEGA) (felt barrier with the patient, avoidance, misgivings about prognosis). Treated as subscales, reliability analysis revealed the following alpha coefficients for the diabetes case: Caring/Supportive Approach, 0.91; Nursing Satisfaction, 0.75; Authoritarian Stance, 0.65; and Negativity, 0.78. For the mental illness case the following alpha coefficients were observed: Caring/Supportive Approach, 0.91; Nursing Satisfaction, 0.75; Authoritarian Stance, 0.73; and Negativity, 0.85. Coefficients are also represented in Table 2 indicating moderate to high level of internal consistency among scale items.

**Table 2:** Pattern matrix showing the final five component solution

	Factor 1	Factor 2	Factor 3	Factor 4	Factor 5	**h**^**2**^
D14 'give more treatment explanation'	**.83**	.04	.15	-.11	.06	.68
M14 'give more treatment explanation'	**.72**	.09	.01	-.22	**-.32**	.74

D15 'explain more, rules regulations'	**.80**	.04	.09	.13	.17	.65
M15 'explain more, rules regulations'	**.68**	.06	.06	-.21	-.27	.65

D16 'ask more about his health'	**.81**	-.02	.10	.10	.10	.66
M16 'ask more about his health'	**.69**	-.05	.05	-.07	-.22	.63

D23 'encourage to discuss concerns'	**.78**	-.12	-.17	.03	.01	.66
M23 'encourage to discuss concerns'	**.64**	-.12	-.10	-.08	**-.30**	.63

D22 'I would support carer more'	**.78**	-.05	-.16	.11	.02	.65
M22 'I would support carer more'	**.65**	-.01	-.13	-.03	**-.32**	.65

D21 'encourage relatives to be supportive'	**.75**	-.01	-.11	.13	.06	.58
M21 'encourage relatives to be supportive'	**.65**	-.05	-.04	.01	-.24	.59

D7 'encourage more, self care'	**.72**	.11	.11	.01	.08	.50
M7 'encourage more, self care'	**.50**	.09	.10	-.15	-.17	.35

D13 'take more gentle approach'	**.59**	-.05	.02	.28	.02	.47
M13 'take more gentle approach'	**.43**	-.12	-.02	.13	**-.44**	.56

D5 'is a challenge'	-.04	**-.80**	-.04	.07	.02	.63
M5 'is a challenge'	-.12	**-.78**	.06	-.12	-.06	.62

D1 ' special skills'	.03	**-.74**	.09	.24	.17	.59
M1 'special skills'	-.03	**-.78**	.05	-.03	-.03	.59

D2 'monotonous'	.07	**.60**	.25	-.01	.03	.44
M2 'monotonous'	.01	**.55**	.12	.24	.12	.41

D3 'special effort'	**.36**	**-.47**	-.09	-.07	-.11	.44
M3 ' special effort'	**.36**	**-.41**	-.01	.07	.27	.37

D10 'not trust his treatment opinion'	-.13	-.10	**.72**	.26	.10	.57
M10 'not trust his treatment opinion'	-.07	.11	**.57**	-.09	**-.38**	.53

D8 'enforce doctors' orders'	.00	-.04	**.64**	.10	.12	.41
M8 'enforce doctors' orders'	-.07	-.02	**.61**	-.09	-.19	.42

D11 'hesitate give early leave'	.09	.06	**.57**	.07	.08	.36
M11 'hesitate give early leave'	-.03	-.02	**.52**	-.14	**-.44**	.50

D9 'doubt his ability, self care'	.26	.22	**.52**	-.06	.06	.40
M9 'doubt his ability, self care'	.09	.29	**.40**	-.07	**-.42**	.54

D20 'I feel barrier between us'	.09	.14	.03	**.72**	-.07	.63
M20 'I feel barrier between us'	.00	.14	-.01	**.34**	**-.61**	.63

D31 'reluctant develop care plan with him'	-.05	-.30	-.06	**.61**	-.07	.52
M31 'reluctant develop care plan with him'	-.10	.31	.16	.19	**-.52**	.55

D19 'avoid confrontation'	.19	.01	-.10	**.55**	.19	.47
M19 'avoid confrontation'	.10	.02	-.12	**.32**	**-.53**	.50

D30 'am not positive about his prognosis'	-.01	-.04	.28	**.56**	-.04	.39
M30 'am not positive about his prognosis'	-.02	.10	.19	.23	**-.34**	.29

D24 'encourage that he talk about problem'	.22	**-.41**	.26	**-.37**	.10	.46
M24 'encourage that he talk about problem'	.13	**-.39**	**.30**	**-.41**	.07	.44

D28 'expect him to be demanding'	-.03	-.12	.26	**.43**	-.27	.38
M28 'expect him to be demanding'	.15	.04	.18	.01	**-.68**	.64

D29 'regard that he require more privacy'	-.04	-.13	.03	**.44**	**-.30**	.35
M29 'regard that he require more privacy'	.08	-.08	-.07	.05	**-.69**	.52

D33 'more patient with him'	**.37**	-.24	-.05	**.41**	-.25	.54
M33 'more patient with him'	**.30**	-.03	-.04	.10	**-.59**	.60

D18 'keep my private life secret'	**.32**	.12	.13.	**.41**	.03	.35
M18 'keep my private life secret'	.34	.06	.11	.09	**-.45**	.49

D34 'ease off on touchy issues'	.29	-.04	-.02	.17	**-.33**	.32
M34 'ease off on touchy issues'	.23	.-02	-.05	.04	-.57	.48

Percent variance	24.9	12.2	6.0	4.7	4.1	Total variance = 51.9%

**Cronbach's alpha D**^**1**^	**.91**	**.75**	**.65**	**.78**	-	

**Cronbach's alpha M**^**1**^	**.91**	**.75**	**.73**	-	**.85**	

^1^D = diabetes case; M = mental illness case

Turning to items excluded from the subscales, as shown in Table 3, the item 'worry about aggression' correlated significantly with a number of factors including Caring/Supportive Approach, Authoritarian Stance and Negativity and this was consistent across patient type. These correlations indicate the lack of unique influence of any dimension and support the exclusion of this item from scale development. The next three items shown in Table 3 also show non-unique contribution to subscales. While there are significant loadings on Caring/Supportive Approach they are also correlated with negativity. The strength of the correlation coefficients is low, accounting for their low communalities within the factor analysis. The item 'more careful about his confidentiality' and 'discuss his management with colleagues' showed different associations between the two patient ratings and the factors, accounting for their exclusion from the final solution in the factor analysis. The remaining two items had very low or statistically non-significant correlations with the factors indicating that their variance was poorly explained by the derived dimensions.

**Table 3:** Correlations between subscale scores and the scores on items that were excluded from the subscales

		Caring/Supporting	Nursing Satisfaction	Authoritarian Stance	Negativity
Q17 'worry about his aggression'	Diabetes	.35***	-.11	.32***	.45***
	Mental Disorder	.53***	-.02	.41***	.54***

Q27 'trust relatives in giving medicine'	Diabetes	.29***	.03	.05	.21**
	Mental Disorder	.26***	.04	-.03	.17*

Q25 'allow his visitors to stay longer'	Diabetes	.26***	-.01	-.05	.15*
	Mental Disorder	.22**	.13	.02	.18**

Q32 'more careful about his confidentiality'	Diabetes	.26***	.07	.09	.22**
	Mental Disorder	.34***	.16*	.22**	.22**

Q4 'discuss his management'	Diabetes	.06	.36***	.08	-.15*
	Mental Disorder	.02	.31***	.11	-.09

Q26 'caution discussing him with visitors'	Diabetes	.05	.00	.17*	.01
	Mental Disorder	.06	.07	.23**	.10

Q12 'expect he will follow instructions'	Diabetes	.12	-.03	.12	.00
	Mental Disorder	.13	-.05	.05	.00

* p < 0.05 ** p < 0.01 *** p < 0.001

### Discriminant Validity

**Figure 1:**
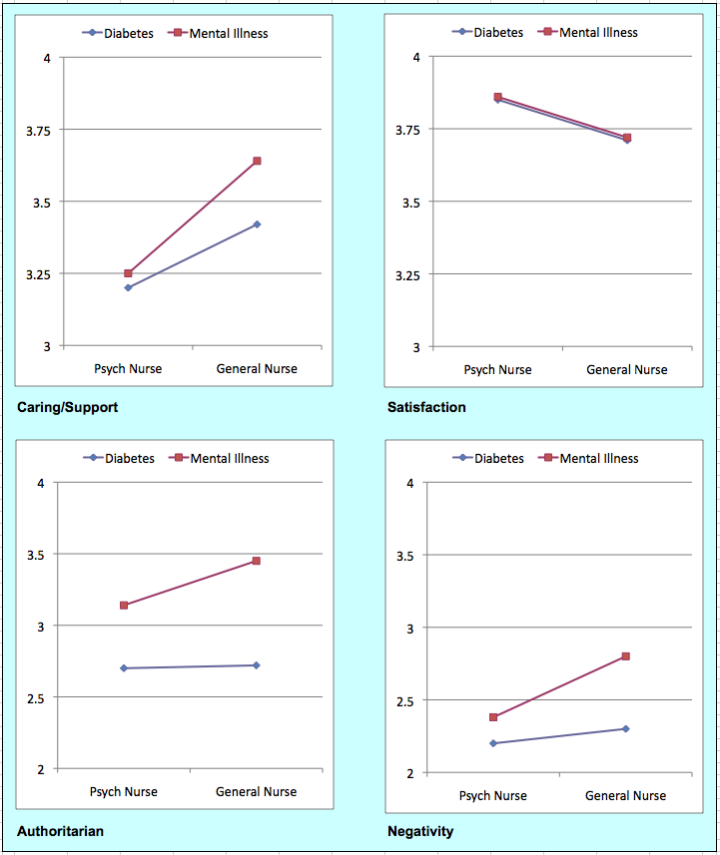
**Mean subscale scores by nurse type and patient type**.

Analyses of variance comparing nurse types (general versus psychiatry) and patient types (diabetes versus mental illness) were conducted to assess the discriminant validity of the subscales of the NRS. Results are summarized in Figure 1. Inspection of the mean scores in the first panel indicates that nurses endorsed Caring/Supporting approach items with mean scores falling between 'tend to agree' (score of 3) and 'agree' (score of 4). As can be ascertained by inspection of Figure 1, general nurses endorsed Caring/Supporting Approach items more highly than did psychiatric nurses and this was verified by a significant difference according to nurse type (F(1, 206) = 6.65, p < .05). Moreover the effect of patient type was statistically significant (F(1,206) = 7.7.1, p < .01) but not the interaction suggested in Figure 1 (F(1,206) = 2.92, p = .09).

Inspection of the means in Figure 1 related to Nursing Satisfaction scores suggest that nurses endorsed high level of satisfaction in the nursing of the patients described in the two vignettes. Scores are near a value of four representing the scale descriptor 'agree'. Statistical analysis revealed no differences according to nurse type (F(1,206) = 2.29, p < .13), patient type (F(1,206) = .26,p = .61) or with respect to the interaction term (F(1,206) = .05, p = .83). Turning to the findings in relation to Authoritarian Stance, the third panel in Figure 1 shows that nurses ranged in their responses from 'tend to disagree' to 'agree' with this approach to the patient, and that this depended on the type of patient considered. The vignette depicting a mental health problem received higher level of endorsement of Authoritarian Stance items than was the case for the diabetes patient. This was reflected in a significant main effect of patient type (F(1,206) = 114.1, p < .001). The main effect of nurse type was not significant (F(1,206) = 2.46, p = .12) however the interaction term was significant (F(1,206) = 7.67, p < .01). As seen in Figure 1 general nurses were more likely than psychiatric nurses to adopt this stance with a mental illness case while they were equally likely to adopt this stance with a case of diabetes.

Finally, Figure 1 suggests that Negativity scores were low in general across all nurses as shown by mean scores ranging between 'disagree' (score of 2) and 'neither' agree nor 'disagree' (score of 3). Again scores appear to depend on nurse type and patient type. Statistical analysis revealed a significant main effect of nurse type (F(1,206) = 10.89, p < .01) with general nurses endorsing Negativity items more highly than psychiatric nurses. In addition, the main effect of patient type was significant (F(1,206) = 78.04, p < .001) with Negativity ratings being higher for the case of mental illness than for the case of diabetes. The interaction term was also significant (F(1,206) = 17.17, p < .001) and, as suggested by inspection of the fourth panel in Figure 1, general nurses were more likely than psychiatric nurses to endorse Negativity items with respect to the case of mental illness. However, this was less so with the diabetes case.

As we have noted there were variations in the mean scores across scales. To demonstrate this, scores from the diabetes and mental illness cases were averaged and a repeated measures analysis of variance was conducted across scales. Results showed a significant main effect of scales (F(1,206) = 160.15, p < .001). As alluded to above, mean scores revealed high endorsement of Care/Supportive Approach (mean = 3.36, s.d. = .89) and Nursing Satisfaction (mean = 3.81, s.d. = .67) intermediate endorsement of Authoritarian Stance items (mean = 2.98, s.d. = .75) and lower endorsement of Negativity items (mean = 2.41, s.d. = .59). Pairwise comparisons of the subscale scores using repeated measures t-tests indicated significant differences for all contrasts at the p < .001 level.

## Discussion

In this work we have begun the development of the NRS, a measure of interpersonal approaches in nursing care. In addition to the application of therapeutic techniques and procedures, the interpersonal relationship in nursing is an important domain for exploration. It is expected that the relationship may determine the level of information patients and families receive regarding medical and nursing procedures, the level and nature of patient involvement in their own treatment, and satisfaction with the nursing and medical contact experience. Moreover, it may be important in nurses' own reflections on their work and in relation to job satisfaction. Examination of the item content of the NRS revealed that item variation could be explained to a great extent by five factors. On further examination the fifth dimension was similar in content to the fourth but reflected rating differences between approaches to nursing patients with mental illness and those with diabetes. As a result we arrived at four conceptual dimensions, which we labeled Caring/Supportive Approach, Nursing Satisfaction, Authoritarian Stance and Negativity.

The NRS was designed to measure key elements of the nurse-patient interpersonal encounter. We anticipate that the availability of the NRS will allow expansion of research into the practice of nursing including variations across nursing settings and different illnesses. In addition a scale of this kind may encourage research into nursing practice as it may be affected by demographics and psychosocial variables of both nurse (e.g., age, gender, level of nursing experience) and patient (e.g., age, gender, education level). In the present paper we present an account of the development of the NRS, its dimensional structure, the internal reliability of its subscales and their discriminant validity. With respect to discriminant validity we examine the ability of the derived subscales to distinguish between responses given by psychiatric and general nurses when considering two types of patients, those with diabetes and those with mental illness.

With the NRS we have attempted to cover both positive and negative nursing actions, particularly as our own background is in mental health where stigma associated with mental illness may influence negative attitudes in health care professionals, including nurses (e.g., Bray, 1999). Such aspects may also be important in the nursing of other conditions that are stigmatised in the general community, such as HIV-AIDS (e.g., [20,21]) or for conditions where nurse training is limited, as in the phenomenon of parasuicide (e.g., [22,23]).

The Caring/Supportive Approach dimension parallels that reported by Lea et al [14]. In addition, it is part of general considerations regarding the notion of care [11,16,24,25] and forms part of conceptualisations of the interpersonal competence of nursing [10]. While the remaining dimensions have not been measured with a closed-ended questionnaire format in previous research, the notion of an Authoritarian Approach to the patient is highlighted in several views of the nursing relationship, including the model suggested by Wade [25]. According to this view, two underlying dimensions describe several styles of nursing. The first dimension is called 'open versus closed' and the second is 'person-centred versus task-centred'. Similar to the Authoritarian Approach identified here, 'closed' relationships involve restricting patient choice, limiting the involvement of visitors, and fostering limited therapeutic input from the patient. In general, this approach is considered to have the potential to damage the therapeutic alliance between patient and nurse (Davies et al, 1997) and direct assessment of patient views has indicated patient dissatisfaction with nurses behaving towards them in this measure [11]. Of interest to some models of the nursing relationship is the issue of nurses' self-disclosure which is incorporated within a single dimension of nursing interpersonal competence [10]. The results from the present study partly dispute this, suggesting that self-disclosure may be part of a separate dimension, Negativity, that is negatively correlated with the Caring/Supportive Approach dimension. Negativity appears to have parallels to the concept of 'distancing' identified by Bray [26] within mental health nursing settings where challenging patient behaviour appears to be associated with greater distancing strategies. The availability of the Negativity subscale within the NRS may encourage further research into this dimension of nursing. Similarly Nursing Satisfaction can further be explored by use of the NRS. This subscale may need extension in future work to separate its two underpinning constructs, 'perceived challenge' and 'satisfaction' with the nursing task.

Turning to the remaining findings, with respect to subscale scores, general nurses endorsed items from the Caring/Supportive Approach dimension more highly than did psychiatric nurses. This was the case for both of the cases (diabetes and mental illness) described in the vignettes. Explanations of this variation need to consider that the items on the scale reflect extra support and care behaviours relative to other patients. Consequently one explanation of the difference between general and psychiatric nurses' responses is that psychiatric nurses relative to general nurses, by virtue of their training and role, already engage in such care processes and do not perceive a need for extra care in their approach to nursing the cases depicted. Both patients in the vignettes suffered from complicated illness, encumbered by psychosocial disability and potential for aggression. Such attributes may involve greater emphasis on approaches that encourage greater support of the patient, and, also the support of others such as family and caregivers beyond the treatment environment.

Nursing Satisfaction scale scores were found to be high regardless of patient type and nurse type. Scale scores reflected a combination of perceived challenge and interest in the nursing task related to the two cases presented. It would seem from their responses that nurses, in the face of illness that is complicated by accompanying psychosocial difficulties and disability, adopted a positive approach to the management of patients (diabetes and mental illness) in vignette format.

Responses to the vignette depicting mental illness differed according to nurse type with respect to adopting an Authoritarian Stance and in relation to Negativity. Perceptions of greater personal lack of control in the case of mental illness relative to diabetes may underpin these differences in responses. Authoritarian Stance items reflect a lack of trust in self-care by the patient and the need to enforce treatment and control regardless of the patient's attitude. General and psychiatric nurses indicated lower level of Authoritarian Stance in relation to the diabetes case than to the mental illness case. In addition, general nurses were more likely to endorse an authoritarian approach for the case of mental illness than their psychiatric counterparts. The relative lack of psychiatric training, relative absence of training in alternative management approaches to such patients, and lack of experience in working with psychiatric patients may have led general nurses to endorse an authoritarian approach more so than psychiatric nurses. In view of this explanation we examined the correlation, post-hoc, between a measure of contact with psychiatric patients and Authoritarian Stance scale scores. This revealed the expected negative association (r(207) = -0.19, p < .01).

Similarly, the study indicated a greater endorsement of Negativity items in relation to mental illness than diabetes and this trend was more prominent in the general nurses than the psychiatric nurses. Negativity items were characterised by a perceived personal barrier with the patient (including the need to preserve this) and greater reluctance to work with the patient using a collaborative relationship. While mental illness attracted higher negativity ratings than diabetes across all nurses, psychiatric nurses were less likely to discriminate between the case of diabetes and the case of mental illness compared with general nurses. Again, the findings bring into perspective the possible effects of lack of psychiatric training and psychiatric exposure in general nurses which, it would appear, may lead them towards avoidance of an otherwise useful nursing care strategy. The role of stigma attached to mental illness (and perhaps other stigmatised patients such as those with HIV-AIDS [20,21] need to be explored in relation to how this may influence nursing approaches. Consistent with this, post-hoc analysis within our own study sample, a measure of negative attitudes towards psychiatric patients was positively correlated with the Negativity subscale (r(207) = 0.5, (p < .001). Also, in view of the possible explanation that lack of training and exposure may underpin higher Negativity, the measure of contact with psychiatric patients was negatively associated with Negativity (r(207) = -0.26, p < .001).

### Strength and limitations

In the present work item responses were directed to cases presented in vignette format. This is a useful approach to comparing perceptions of the nursing role across different illnesses and across nurses with differences in education and clinical experience. Across a variety of patients and nursing roles it is possible to identify, as we have begun to do here, common dimensions in the interpersonal behaviours of nurses as well as differences in those behaviours. However, there is a need to extend this work to perceptions of behaviours of nurses in the treatment of actual patients to accommodate variations that derive from day-to-day patient management. The NRS may also be used to explore how patient management may vary between hypothetical cases and real practice with actual cases by combining vignette and direct interaction information. This may help to explore how pre-conceptions may be modified by direct patient exposure. There is also work to be done in relation to the possible extension of the NRS to capture additional dimensions. In the present study we excluded a number of items that did not fit the factor structure of the NRS and there is a strong possibility that for some of these their lack of fit may represent under-sampling of items from dimensions not well measured by the present version of the NRS. Furthermore there is a need to examine test-retest reliability of the scales despite our demonstration of high internal consistency among the items.

## Conclusions

Previously developed measures on nursing care have included aspects of the nursing relationship but are not focused on the issue of nursing a specific type of patient. They are also focused on broader constructs of nursing care, including technical duties and professional roles, and not on the interpersonal relationship between the nurse and the patient.

The development of the NRS provides a measure of four key dimensions of the nursing relationship that may contribute to a clearer understanding of the difference in nursing practice approaches towards particular types of patients, and may be particularly useful in survey research with large samples of nurses. The four dimensions for which reliable scales were derived were Caring/Supportive Approach, Nursing Satisfaction, Authoritarian Stance, and Negativity. A scale which measures such items can be used cross-culturally in mapping attitudes and practice development, quality improvement and nursing training.

## Competing interests

The authors declare that they have no competing interests.

## Authors' contributions

TKK carried out the research as part of a Master program under the supervision of the late Steven Klimidis, with co-supervision and input from HM. TKK prepared the first draft of the paper which was substantially modified and completed by HM. Both authors have approved the final version of the manuscript.

## Supplementary Material

Additional file 1 **Questionnaire items and vignettes**. The additional file contains the vignettes that were used in the study and the 34 items of the Nursing Relationships Scale. Click here for file
