# Supplementary material for: Development of the Nursing Relationships Scale: a measure of interpersonal approaches in nursing care
Source: Int J Ment Health Syst. 2010 May 28;4:12. doi: 10.1186/1752-4458-4-12 (PMC2889861; doi:10.1186/1752-4458-4-12)
Supplement: Additional file 1 — Questionnaire items and vignettes. The additional file contains the vignettes that were used in the study and the 34 items of the Nursing Relationships Scale. [file 1752-4458-4-12-S1.PDF]

Additional File 1: Questionnaire items and vignettes

The Nursing Relationship Scale and case vignettes used in this study. The vignettes preceded answering the 34 questions of the NRS. Item numbers correspond to those shown in the Tables within the body of the article.

Read the following description of Mr. Jones who suffers from diabetes.

Mr Jones is a 22 years of age and lives at home with his parents. He has suffered from diabetes for the last two years and his condition has not been under sufficient control for most of this period. After the onset of his illness he has held only a few temporary jobs and is now unemployed. He has progressively lost his self-confidence and has tended to stay in his room at home and not do much around the house. His parents say that his temper has been increasingly bad over the last three months. Nevertheless, he remains in contact with a few friends but has tended not to go out as much as he used to.

Read the following description of Mr Smith who suffers from a mental illness.

Mr Smith is 24 years of age and lives at home with his parents. He has had a few temporary jobs since leaving school but is now unemployed. Over the past six months he has more or less stopped seeing his friends and spends most of his time in his room at home. He neglects to do chores at home and when his parents request this from him he loses his temper easily. Even though he is alone in his room his parents have often heard him arguing as if someone else is there. When his parents have encouraged him to go out he says he won't leave home because he feels that someone is spying on him.

Please answer the following questions regarding how you might work with Mr J/S if he were your patient in the ward. To what extent do you agree with the following statements in the management of Mr J/S.

1. Looking after Mr J/S would be very satisfying to me because his management requires very special skills.
2. Working with Mr J/S would be monotonous and too routine.
3. I would make a special effort to look after Mr J/S.
4. I would discuss Mr J/S's management with my colleagues in the ward.
5. Looking after Mr J/S would be a challenge that I would look forward to.
6. I would spend extra time with Mr J/S than with other patients in the ward.
7. I would encourage Mr. J/S, more than other patients, to take care of himself as much as possible.
8. If Mr J/S refuses medication I would try to enforce 'doctor's orders'.
9. I would have some doubt that Mr J/S could contribute significantly to his care plan.
10. I would not completely trust the opinion of Mr J/S in making treatment decisions.
11. If Mr J/S, soon after his admission, requests weekend leave I would be hesitant to authorise this.
12. I would expect that Mr J/S would follow my instructions regarding his treatment regime.
13. I would be more 'gentle' in my approach to Mr J/S than I would with other patients in general.
14. I would take care, more so than usual, to provide Mr J/S with an explanation about a nursing action or treatment.
15. I would take care, more so than usual, to explain to Mr J/S about the ward's rules, regulations and general culture.
16. I would take care, more so than usual, to ask Mr J/S about his state of health.
17. I would worry, a little more so than usual, that Mr J/S may become aggressive in the ward.
18. Compared with other patients, I would avoid letting Mr J/S know about me or my private life.
19. Compared with other patients, I would avoid confronting Mr J/S if he did something against the rules of the ward.
20. I would feel a 'barrier' between me and Mr J/S, more so than with other patients.
21. Compared with other patients, I would encourage the caregivers of Mr J/S to be supportive.
22. Compared with other patients, I would be very supportive to the caregivers of Mr J/S.
23. More so than usual, I would ask Mr J/S if he would like to discuss any problems or concern he is having about his stay in hospital.
24. I would make an effort to encourage Mr J/S to talk about his problems or illness.
25. If a visitor of Mr J/S would try to stay beyond visiting hours I would allow this.
26. I would be cautious about discussing Mr J/S's condition with any of his visitors.
27. I would trust Mr J/S's relatives in administering medications.
28. I would expect that Mr J/S would be a more demanding patient than most.
29. I would regard that Mr J/S requires more privacy in the ward than most other patients.
30. I would not be too positive about Mr J/S's prognosis.
31. I would be a little reluctant to work together with Mr J/S to develop the care plan.
32. I would be especially careful about confidentiality regarding Mr J/S's condition.
33. I would be more patient with Mr J/S than with other patients in the ward.
34. I would be more reluctant than usual to persist with an issue that Mr J/S does not want to discuss.
